# Delicar: A Smart Deep Learning Based Self Driving Product Delivery Car in Perspective of Bangladesh

**DOI:** 10.3390/s22010126

**Published:** 2021-12-25

**Authors:** Md. Kalim Amzad Chy, Abdul Kadar Muhammad Masum, Kazi Abdullah Mohammad Sayeed, Md Zia Uddin

**Affiliations:** 1 Department of Computer Science and Engineering, International Islamic University Chittagong, Chittagong 4210, Bangladesh; kalim.amzad.chy@gmail.com (M.K.A.C.); akmmasum@iiuc.ac.bd (A.K.M.M.); s.titas244@gmail.com (K.A.M.S.); 2 Software and Service Innovation Department, SINTEF Digital, 0316 Oslo, Norway

**Keywords:** computer vision, self-driving car, smart product delivery, Internet of Things, convolution neural network, Raspberry Pi 3

## Abstract

The rapid expansion of a country’s economy is highly dependent on timely product distribution, which is hampered by terrible traffic congestion. Additional staff are also required to follow the delivery vehicle while it transports documents or records to another destination. This study proposes Delicar, a self-driving product delivery vehicle that can drive the vehicle on the road and report the current geographical location to the authority in real-time through a map. The equipped camera module captures the road image and transfers it to the computer via socket server programming. The raspberry pi sends the camera image and waits for the steering angle value. The image is fed to the pre-trained deep learning model that predicts the steering angle regarding that situation. Then the steering angle value is passed to the raspberry pi that directs the L298 motor driver which direction the wheel should follow. Based upon this direction, L298 decides either forward or left or right or backwards movement. The 3-cell 12V LiPo battery handles the power supply to the raspberry pi and L298 motor driver. A buck converter regulates a 5V 3A power supply to the raspberry pi to be working. Nvidia CNN architecture has been followed, containing nine layers including five convolution layers and three dense layers to develop the steering angle predictive model. Geoip2 (a python library) retrieves the longitude and latitude from the equipped system’s IP address to report the live geographical position to the authorities. After that, Folium is used to depict the geographical location. Moreover, the system’s infrastructure is far too low-cost and easy to install.

## 1. Introduction

Failure to deliver the product in time is a typical scenario of Bangladesh that affects the economy significantly. Among different reasons, the root cause of this scenario is to stay stuck in traffic congestion. According to a recent statistic, because of the congestion in Dhaka, the capital of Bangladesh, the amount of loss is around BDT 200 billion annually [1]. Investigators have reported a loss of 3.2 million working hours a day of traffic jams [2]. The Center for Economics and Business Research is projected that, by 2030, it will increase to almost BDT 300 billion [2]. Furthermore, in our country, road accidents are deeply linked with drivers’ behavior. Most of them are tempted to race on the lane, neglecting the risk of an accident. Disobeying traffic regulations and signals also leads to critical accidents and disasters. This ill-mindedness has caused so many disasters, taken too many souls and caused mass destruction in the last decades across the world. At least 4138 people were killed and 4411 wounded in 4147 crashes in 2019, while 2635 were killed and 1920 wounded in 2609 accidents in 2018, according to police [1]. In cases where it is impossible for a person to avoid a car accident, self-driving cars will save millions of lives and subside the on-time product delivery failure case without road accidents.

Artificial Intelligence (AI) plays a significant role in almost every aspect of human life, in every type of industry. For example, researchers [3,4] used a support vector regression algorithm to predict the water parameters. Considering physical and operational factors, another group of researchers [5] engaged AI to assess pipe break rate and [6] decoding clinical biomarker space of COVID-19. Nowadays, AI is also broadly used in building the smart city [7,8], smart meter [9,10], agriculture [11,12,13], education [14,15], healthcare [16,17,18] and so on. Machine learning is a branch of artificial intelligence that allows machines to learn without being explicitly taught from prior data or experiences. Nowadays, the neural network is a popular type of machine learning algorithm that mimics the human brain. CNN (Convolutional Neural Networks) and other groundbreaking systems have provided tremendous results in computer vision. In the majority of cases, they improved the preceding manual extraction features and created new cutting-edge solutions for such tasks as image classification [19], captioning [20], object detection [21] or semantic segmentation [22]. A machine’s reaction times and alerts are far better. In addition, these vehicles were fitted with extraordinary capabilities by long-range cameras and ultrasonic sensors. Since the last decade, extensive work has been carried out on autonomous robotics and driving systems. Many research studies focus on the classification, identification and development of decisions based on the vision to improve, evolving techniques and algorithms. There are also some off-road studies. In our comprehensive study, we have felt the need for some missing features or works in those studied works.

Our self-driving product delivery vehicle can move on a road autonomously through the deployed deep learning pre-trained model. The car’s key input is real-time camera footage mounted on the roof. The system outputs the respective steering angle and drives the car accordingly. Because the camera is the only control system input, the purpose of the project is to teach the vehicle how to handle the steer. The network is trained on a different machine and then shifted to an onboard computer to regulate the vehicle. Then the autonomous product delivery vehicle is entirely independent of other machines. Furthermore, the position of the car is reported to the authority through a map to monitor. Obstacle avoidance is a different problem that can also be overcome, but it goes outside the scope of the study to combine it with the system. The current system configuration is not that capable of dealing with both steering angle prediction and obstacle avoidance. This self-driving vehicle work will significantly change traffic systems and public safety in a developing country like ours. It can also support national defense forces to perform ground monitoring or conduct rescue tasks. More particularly, the risk of an accident can be reduced dramatically. Moreover, the development cost of this system requires about BDT 30K–40K for hardware and 20K–30K for software and other experimental purposes. As a result, product delivery car owners in developing nations like Bangladesh would find the technology beneficial and economical.

The objectives of this research are to develop a self-driving car for overcoming the product delivery failure without any road accidents, to design a low-cost infrastructure with effective outcomes, to build an end-to-end deep learning model equipped in the self-driving car prototype, and to broadcast the geographical location of the vehicle through a map in real-time.

With the introduction, this paper is composed of five parts. Section 2 covers the literature review, and Section 3 contains working procedure, functional units, dataset collection, normalization, augmentation, pre-processing, deep learning model and driving instruction forwarding strategies. Section 4 shows the experimental outcomes. Finally, Section 5 addresses the analysis and future scope.

## 2. Related Works

**Figure 1:**
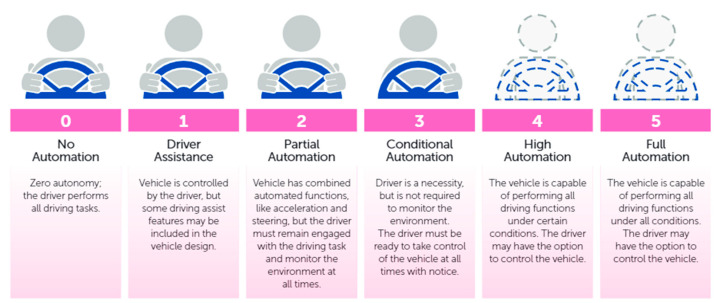
Levels of autonomous driving by NHTSA.

Lots of significant works and research have been performed on the autonomous vehicle aspect. The NHTSA (National Highway and Traffic Safety Administration) describes five levels of autonomous vehicles [23] shown in Figure 1. In no automation (level 0), the human driver does all the driving. Lane-keeping, cruise control or assisted breaking are a few examples of level 1(driver assistance). Tesla Autopilot [24] claims at their level 2 position. The Waymo (Google) self-driving car [25] is an example of conditional automation (level 3). Waymo announced in 2017 that they are testing level 4 driving [24]. Full automation (level 5)—The driving system takes complete control over the entire driving task under all circumstances. The human driver does not need to be inside the car. Recent attacks targeting VANET (Vehicular ad hoc network) with autonomous Levels 1 to 4, which are not entirely autonomous, have been documented. Denial of service attack [26], sybil attack [27], timing attack [28], illusion attack [29], message tampering [30], and node impersonation [29] are examples of these types of attacks.

**Figure 2:**
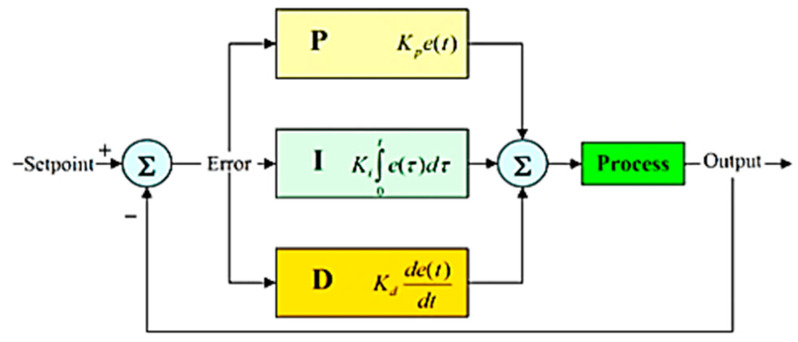
Calculation of correction value in a PID loop.

The non-AI solution practices control theory to determine a steering angle to hold the vehicle on the desired trajectory, typically identified by algorithms for computer vision. PID (Proportional Integral Derivative) controller is one of the most popular methods in control theory [31]. The controller functions in a loop that continually computes an error value *e*(*t*) as a variance between the input from the vehicle and the next command signal. A correction will be measured and applied afterwards. The correction value *u*(*t*) consists of three parts (proportional, integral, derivative) and, as shown in Figure 2, can be determined from the error *e*(*t*).

The whole mathematical formula is the following:

(1) $$u\left(t\right)=k_{p}e\left(t\right)+k_{i}\int_{0}^{t}e\left(t\right)dt+k_{d}\frac{de\left(t\right)}{dt}$$

**Figure 3:**
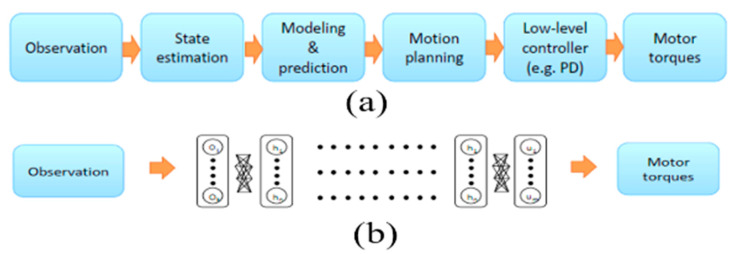
Standard robotics control vs DNN based end-to-end control [10]. (**a**) standard approach (**b**) deep neural network approach.

The standard approach to solving the problem of self-contained driving has divided the problem into several sub-problems, including lane marking, path planning and low-level control, which make up a processing pipeline [32]. Researchers have recently explored a new approach that simplifies the standard control pipeline dramatically through deep neural networks to produce direct control outputs from sensor inputs [33]. The gaps between the two methods are shown in Figure 3. Figure 3a visualizes the standard approach in which the system predicts the motor torques based on the observation of the image data. This approach split the problem into several sub-problems such as state estimation, modeling and prediction, motion planning, low-level controller. In contrast, to solve the same problem, Figure 3b demonstrates a deep neural network approach to predict the motor torques directly from the image observation.

**Figure 4:**
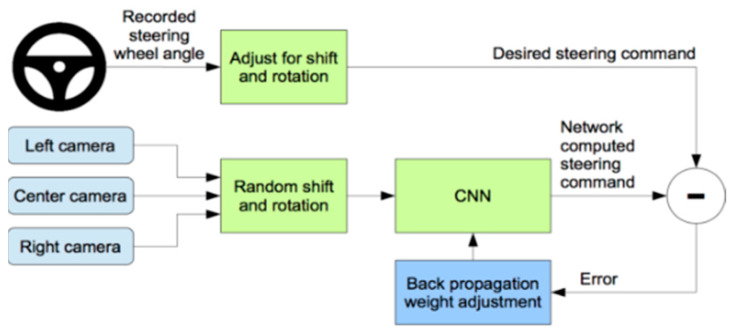
Training the neural network [13].

In the late 1980s [34], a modern, completely linked neural network employed neural networks to monitor automatic cars. In the late 2000’s it was later demonstrated [35] using a six-stage, fully interconnected neural network (CNN) in the DARPA Autonomous Vehicle (DAVE) project and most recently in the NVIDIA DAVE-2 project [32], with a nine-layered CNN network. The training process of the NVIDIA project has been displayed in Figure 4, where the steering angle is recorded for the center camera image and the left and right camera image steering angle is shifted. Then fit into CNN architecture, calculate the error and adjust the weight via backpropagation. The architecture of the CNN model used by NVIDIA is nine-layer depth, including 5 convolution layers and three dense layers. The first three convolution layers contain 24, 36, 48 kernels and the rest two convolution layers consist of 64 kernels. This architecture includes 27 million connections and 250 thousand parameters.

**Figure 5:**
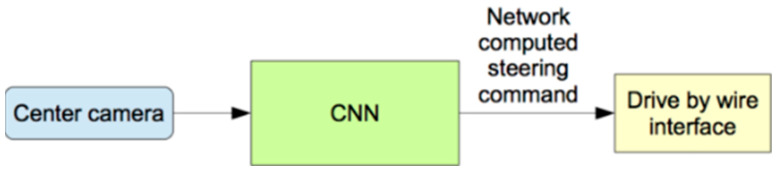
Testing the neural network.

The testing procedure (Figure 5) is a sample where the weight and the CNN architecture are saved, and the camera image goes through that saved model, predicting the steering angle and the car drive by the wired interface.

There are two different phases to the use of deep neural networks [36]. The first step is training, in which the backpropagation techniques change the weights of the network. The next phase is when unseen data are fed into the network to produce the predicted output (e.g., the predicted image classification, for example) once it has been trained–i.e., network weights minimize errors in training example. The training phase is generally more computational and requires high throughput, usually not available on embedded platforms. On the other hand, the inferencing process is comparatively less computer-intensive and latent, if not more so, is as critical as software output because many case stores have strict real-time requirements. For example, with neural network and computer vision-based learning methods, Masum et al. [37] attempted to introduce an autonomous automotive program. The system predicts the steering angle learning from live images according to which the vehicle moves autonomously.

David Stavens et al. [38] have attempted to describe the ruggedness of autonomous off-road vehicles for the terrain project. They proposed a supervised machine learning approach to estimate the roughness of the terrain from laser range data. They used data from the 2005 DARPA Grand Challenge to compare nearby surface points acquired with a laser. Bajracharya et al. [6] did the same kind of work in their research. They used self-supervised training from sensors to know the near-field terrain traversability. The near-field classification was then used to direct the far-field training of terrain traversability. As part of the DARPA Learning Applied to Ground Robots (LAGR) project, the methodology developed was incorporated into a fully autonomous off-road navigation system. Problems in mobile off-road vehicles and mobile robotics caused by poor stereo vision are increasing and remain vulnerable for as long as possible. Junsoo Kim et al. [39] introduced a model focused on long-distance stereo vision to solve this problem. Training data generation on every image frame in a self-supervised way gives robust, consistent stereo module label input, ensuring success. From an input image, meaningful features are acquired, and information is learned. These features train real-time classifiers that can identify complex terrain to distance from the horizon. They claim that it exceeds the max stereo range of 12 m and can see paths and obstacles at a distance of 5 to more than 100 m [39].

The extensive usage of self-driving technology is exemplified by trains [40]. Some of such self-driving trains include the Docklands Light Railway (DLR) in London, UK [41], Yurikamome in Tokyo, Japan [40], London Heathrow airport’s ultra-pods [41] and SkyTrain in Vancouver, Canada [42]. The successor of Robot Operating System (ROS) ROS2 based self-driving vehicle architecture can activate safe and reliable real-time behavior [43]. Bakioglu et al. [44] proposed VIKOR and TOPSIS algorithms to prioritize risks in self-driving cars, while another group of researchers [45] proposed a self-driving delivery robot in last-mile logistics. Navigation routes, one-way streets, speech recognition, and no-entry status are all things that self-driving vehicles require [46]. Based on an adaptive large neighborhood algorithm (ALNS), Guo et al. [47] proposed a multimodal transport distribution model for self-driving vehicles. Dommès et al. [48] investigated aged and young pedestrians’ behavior in front of the conventional and self-driving car wherein mixed traffic conditions. They undertook the simulated two-way street-crossing task. When delivering commands to a self-driving vehicle Deruyttere et al. [49] developed a model that can determine uncertainty, detect the causing objects of uncertainty and generate a question for the passenger that describes the objects.

Tinghui Zhou et al. [50] used monocular video sequence networks of a single view depth and multi-view pose. They approached it as unsupervised through similar approaches were made by others as supervised. Using the images, they were tempted to train the network with a targeted view (single view) and computed losses from some multi-views (closer and distant views from the target view). Yanlei Gu et al. [51] proposed a prototype mimicking the human driving system from the actual traffic environments dataset. Again, different algorithms for different functions of the autonomous vehicle have been suggested. Such as Voronoi Diagram (complete but limited to the static environment), Occupancy Grid (low computational power but has problems vehicle dynamics), Driving Corridors (continuous collision-free space findings but costs of computation with motion constraints), etc., algorithms are used for planning for searching the best space available in the path. Driving Corridors and Non-Linear Constrained Optimization method for intersection and Multiple Criteria Decision Making for non-intersection segments planning, Mixed-Observability MDP for pedestrian crossing, etc., these implemented obstacle detection and decision making. Trajectory Planning is being worked out by Tiji Algorithm, 4th Order Polynomials, Cubic Bezier curves, etc., and many other algorithms are used [52]. Here the authors provided elaborated criticism and evaluation of such algorithms based on different factors.

In manufacturing plants, a line following robot is often used for the pick-and-place features. The robot receives the products from a position and deposits them on an intended location via a pre-specified path. This route is often specified on a black surface as a white line or on a white surface as a black line. Mostafa et al. [53] propose an amphibian line following robot, which can move in both lands and at certain water levels. A line that follows a robot reaches its target by following the predefined path as a white line over a black surface. The line IR sensor is often used to determine which emission led will emit an infrared ray, and the detector led will receive the infrared ray. By a fixed threshold, the robot will sense the rows. L293D motor driver regulates the wheel position and torque of the vehicle. For vehicle rotation, the DC motor is placed onto the wheel. The system can sense the water road and, like a speed boat, activate a propeller mechanism with an integrated water sensor. Since it is an autonomous device, the planned robot is free of any direct human intervention. The idea of the line after the robot is used for different sectors such as rescue, recreation, libraries, searches, and the army. Colak et al. [54] have developed a clever robot line to keep children entertained in shopping malls. This system uses a black line of 4.8 cm to load up to 400 kg. The control functions are remote and manual. Islam et al. [55] have also proposed a low-cost system that can travel around 500 gm without falling off the ground. To strengthen the health care system, Punetha et al. [56] used a robot concept row. If the patient requires drugs, the medicine will automatically be transported along the road, reducing human effort.

A group of researchers [53] proposes an amphibian line following robot for product delivery in Bangladesh perspective, which can move in both lands and at certain water levels. A line that follows a robot reaches its target by following the predefined path as a white line over a black surface. However, ensuring a predefined path as a white line over a black surface for a long distance is a great challenge for this system. In contrast, our proposed approach can decide the driving direction based on the existing road lane, capturing real-time road images in adverse weather such as rainy, cloudy, etc. In Bangladesh, such a kind of system will be a great addition in ensuring on-time product delivery. Many research studies focus on classification, identification and development of decisions based on the vision to improve, evolving techniques and algorithms. There are also some off-road studies. In our comprehensive analysis, we have felt the need for some missing features or works in those studied works. In traffic situations, weather plays an important role. It also influences vision-based independence.

An Extended Kalman Filter (EKF) localization technique considers adverse weather conditions while estimating the car’s posture by registering 3D point clouds against gaussian mixture multiresolution maps [57]. In another study, Ahmad et al. [58] consider weather and lighting conditions in the context of road marking. They consider various messages as distinct categories, while most systems [59,60] use OCR-based algorithms to detect letters first and then write. Unlike stormy, rainy days, dark conditions are created and lighting on the bright sunny day. Such changes affect the camera or other sensors for visual input. These environmental effects are considered in various contexts such as estimating the car’s posture, road markings, etc. However, it was not investigated so thoroughly in a dark, rainy environment while the sensor captured image is not clear as it is supposed to be. Land and off-road research primarily illustrated the roughness of the route, visibility and road roughness styles. Very few have examined fragile or damaged road sections (such as deep holes, damaged/broken road pieces, etc.). From a country viewpoint, damaged and broken highways are causing severe traffic and transportation havoc. Studies showing the identification of these broken sections of the road were not possible as well as other cases. Furthermore, the cost of lots of studies is not optimized. Some used Bluetooth modules to communicate and transfer data between vehicle and computer which is expensive and not required. Moreover, using a Bluetooth device reduces the power of a self-driving car’s ability for a long drive as the coverage of a Bluetooth module is very limited. Furthermore, there is no feature to monitor in real-time and observe the geographical location of the vehicle.

## 3. Methodology and Implementation

**Figure 6:**
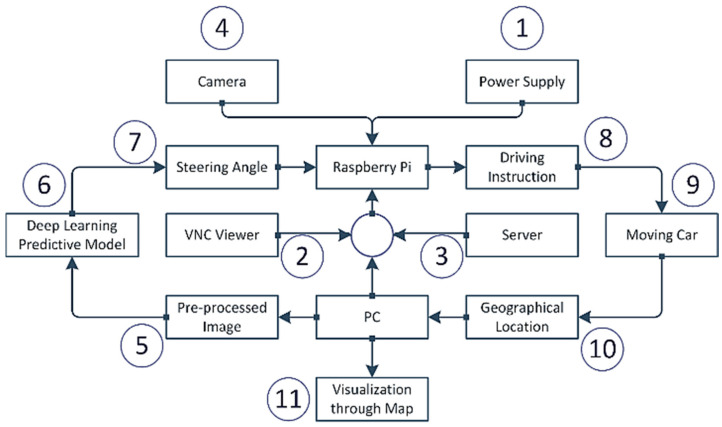
The overall design of the system.

A good design of a system has a significant impact on the successful implementation of a project. The overall architecture of the system is demonstrated in Figure 6. By supplying the power into the Raspberry pi, the heart force of the system, the system starts to initiate. A buck converter converts the 12V lipo battery power supply into 5V and 3A and continuously feeds into raspberry pi enough for raspberry pi to be operating. To program and utilize the raspberry pi despite an extra monitor, we have used a VNC viewer from a local pc. VNC viewer provides instant remote access to the target computer. As the RAM of raspberry pi is too slow to run a pre-trained deep learning predictive model, we need to choose a technique where the predictive model runs into another high-configured computer and the data transfers to the raspberry pi. The high configured local computer acts as a host, the raspberry pi as a client, and the server uses a Transmission Control Protocol (TCP). After establishing the communication between the local pc and raspberry pi, the camera module becomes active and transfers the image to the pc.

Because of the low processing power of the raspberry pi, per second, only ten images have been sent to the local pc. After receiving the image, the image goes through the pre-processing steps that include removing the upper part of the image, blurring the image, transforming the image from RGB to YUV and resizing the image. Then the pre-processed image is sent to the pretrained deep learning model based upon the extended version of the Nvidia CNN model for the self-driving car. The pretrained model can predict what the steering angle for that image in that situation is. The steering angle data is transferred to the raspberry pi through the previously established communication. Based on this steering angle, the raspberry pi decides which direction it should advance, either forward, left, right, or reverse. This instruction is transferred to the self-driving product delivery car. Based on the instruction, the vehicle follows the direction. From the IP address, one can find out the geographical position of the vehicle and track it. Furthermore, the system visualizes the geographical position, i.e., longitude and latitude, through a well-organized map. Each step is discussed in upcoming sections.

This section may be divided into subheadings. It should provide a concise and precise description of the experimental results, their interpretation, and the experimental conclusions that can be drawn.

### 3.1. Functional Hardware Units of the System

To develop the system, we require hardware tools as well as software tools. In our project, we have used different components for controlling speed, direction, transmitting and receiving data, and showing the vehicle’s speed on display. The hardware components used in our project are enlisted below:

1. Raspberry Pi 3 Model B+
2. NoIR Camera with Night Vision
3. Motor Driver IC (L298)
4. Plastic Gear motor
5. 3 cell Lipo Battery (12V)
6. Buck Converter
7. Acrylic Chassis Board
8. Connecting wires
9. Switch

### 3.2. Functional Software Tools of the System

To develop the system, we require software tools along with hardware tools. To drive the hardware, the software performs a leading role. Following software, programming language, library, package, etc., are used in our work:

1. Python programming language: Python is a high-level, general-purpose programming language.
2. Google Colab: Colaboratory (also known as Colab) is a free Jupyter notebook environment running in the cloud and storing on Google Drive notebooks.
3. Numpy: NumPy is a library that supports multi-dimensional arrays and matrices.
4. Pandas: Pandas is used for data manipulation and analysis.
5. Matplotlib: Matplotlib is the Python programming language plotting library.
6. Keras: Keras is an open-source neural-network library written in Python. It can run top of TensorFlow, R, Theano or PlaidML, to allow quick experimentation with deep neural networks [61].
7. Tensorflow: TensorFlow is an open and free software library for data flow used for machine learning applications like neural networks.
8. Imgaug: A library for image augmentation in machine learning experiments, particularly CNN (Convolutional Neural Networks).
9. OpenCV: OpenCV-Python is OpenCV’s Python API. It integrates OpenCV C++ API’s best qualities with Python language.
10. Scikit-learn: It is a free machine learning library for the Python programming language.
11. VNC viewer: VNC Viewer transforms a mobile into a virtual desktop, giving one immediate access from anywhere in the world to one’s Mac, Windows and Linux computers.
12. Sublime Text 3: Sublime Text is an advanced script, markup and prose text editor.
13. Geoip2
14. Folium

### 3.3. Power Supply Strategy

**Figure 7:**
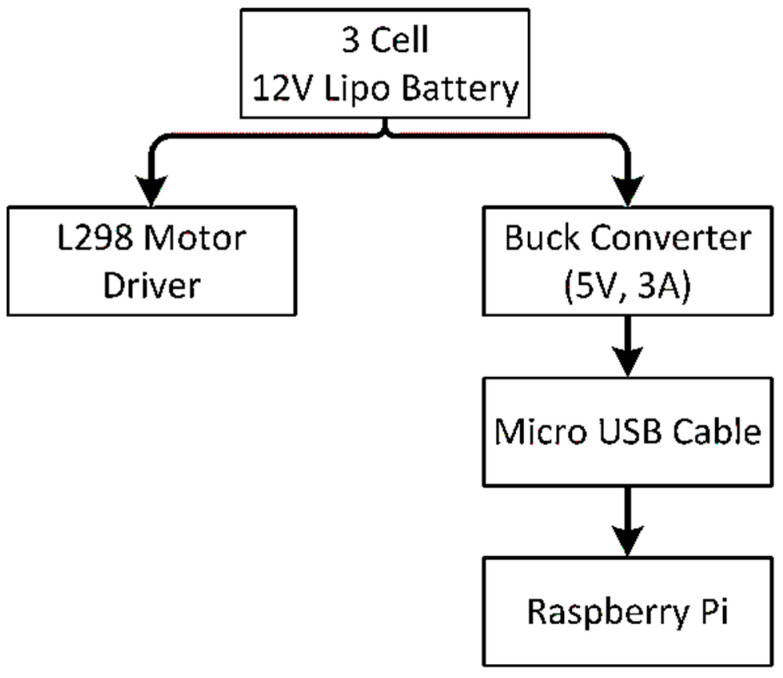
Power flow strategy.

The power supply strategy is displayed in Figure 7. A 3cell 1500 mah 12V Lipo battery is used as the primary power source that supplies the power to the raspberry pi and L298 motor driver. The raspberry pi requires 5V and 3A to come into the working state.

A direct connection with the battery may cause the death of raspberry pi because of the overpowering supply. So, to regulate the power supply, we have placed a buck converter in between lipo battery and raspberry pi that continuously provides 5V and 3A. The raspberry pi connects with the buck converter through a micro USB cable.

### 3.4. Deep Learning Predictive Model

**Figure 8:**
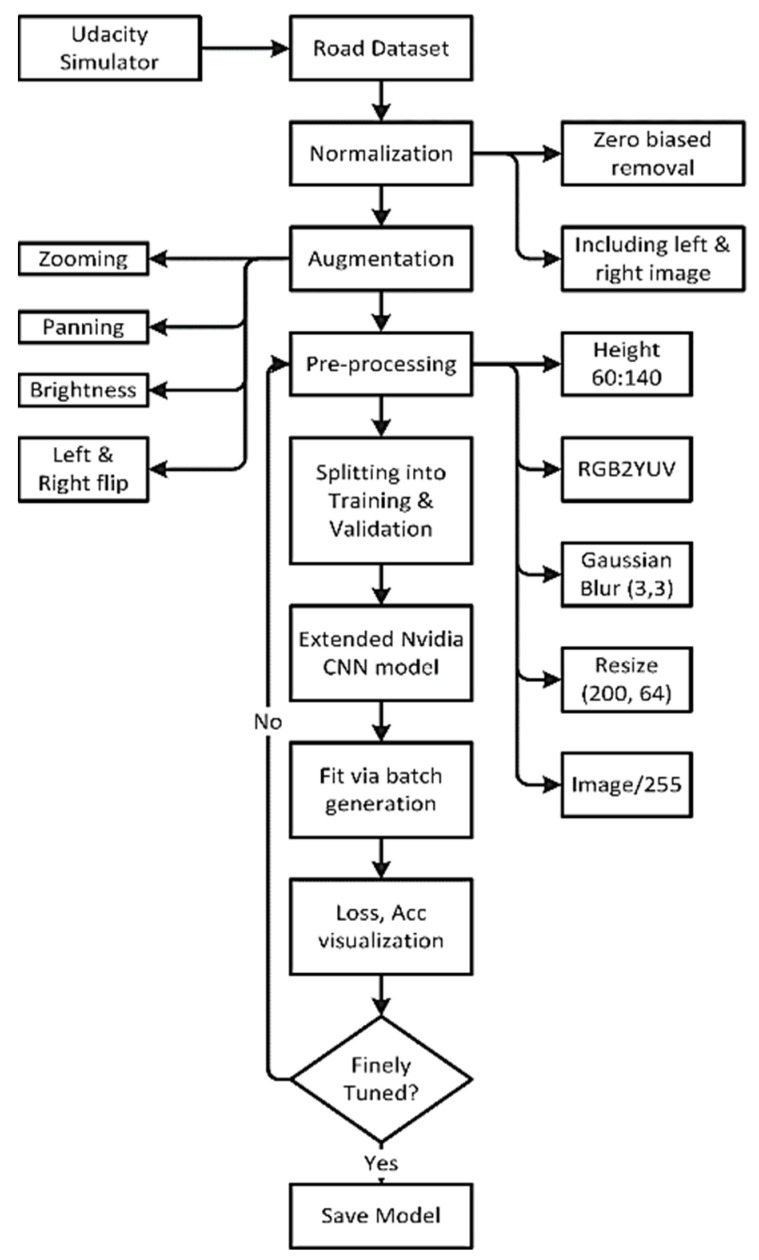
Flowchart of the steering angle prediction model.

Figure 8 is a step-by-step developing process of the predictive model to forecast the steering angle based upon the given road image.

### 3.5. Dataset Collection

We need a dataset containing a massive collection of road images and steering angles against that image for a deep learning predictive model. Different nations’ legislators (e.g., the USA, China, Australia, Singapore, and South Korea) [62,63,64,65,66,67,68] have established or are adopting different regulating measures to enhance the security and privacy of data utilized and sent by autonomous cars. The gathering of data on public roadways is essential for self-driving car autonomy [69]. There exist several datasets developed by individuals or organizations such as Sullychen [70], Nvidia [32], Udacity, commaai, Apollo [71], etc. However, the dataset is too large and beyond our processing capability because of our limited computational resources. For example, the opensource dataset by commaai is 45GB in compressed and 80 GB in uncompressed [72]. In Ref. [73], the authors provide 27 publicly available vehicle datasets, assess them based on various parameters, and recommend selecting the most suited dataset for specific goals. Furthermore, Udacity published a huge open-source dataset in a sunny and overcast environment ranging from 23 GB to 183 GB in size [74]. So, for experimental purposes and considering the limited hardware resources we have developed our own dataset using an open-source Udacity simulator [75]. This simulator was designed for a Nanodegree program of Udacity in a unity environment with two moods. One is training mood and another one autonomous mode. One can drive a car in two tracks, and at the time of driving the steering angle, throttle, speed, etc., is recorded against each image. At the training mood of the Udacity simulator, one needs to set the path directory where the image will be saved and the steering angle is saved as a log file against each image.

We have collected the data on track two and saved the data into a folder shown in Table 1. There have three cameras in the Udacity simulator that track center, left, right images accordingly. Besides steering angle, it also saves the throttle, reverses the speed at that time. The images are saved in jpg format into a different folder, while a log file into CSV format tracks the image path. In this way, we have collected more than 8.4K images based on developing our predictive model.

### 3.6. Normalization

**Figure 9:**
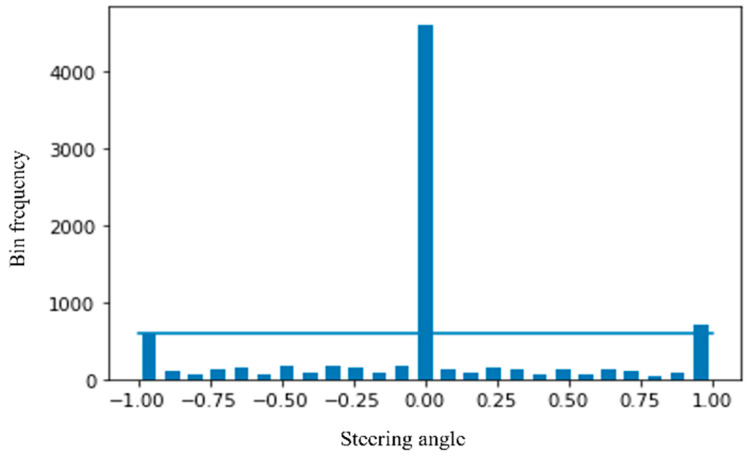
Visualization of the dataset.

To understand the data distribution against the steering angle, we need to visualize the dataset. Through histogram, in Figure 9, we have visualized the data across 25 bins where the zero steering angle is too high, about more than 4K. So, we need to remove zero biased data so that the model generalizes the steering angle.

**Figure 10:**
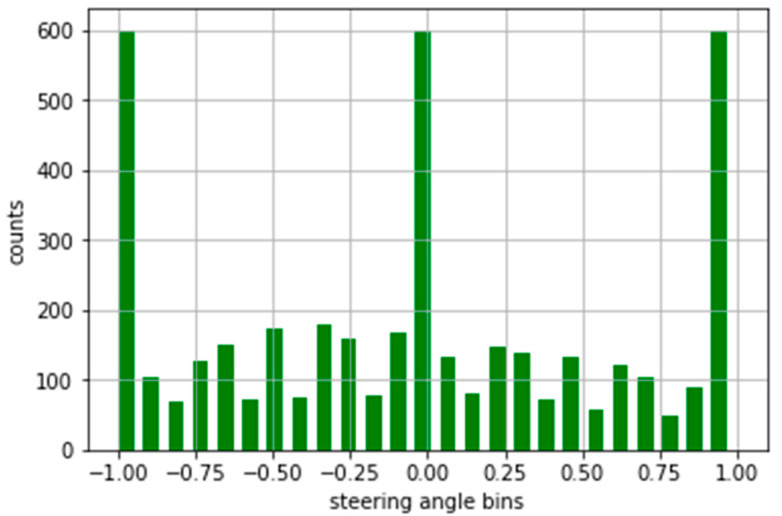
Normalized form of the dataset.

We have considered a maximum of 600 hundred images per bin (Figure 10). So, the more than 600 images bin keeps a maximum of 600 images and removes the rest of the images. After this type of normalization, our dataset is down from 8.4K to 4K, which is too low.

To increase the dataset, we have also considered the left and right images. The steering angle in the dataset is actually based upon the center image. So, the steering angle will be slightly sifted from the center for the left and right images. We have considered 0.15 positive sifted for the left image and 0.15 negative shifted for the right image. Furthermore, the left and right images help us more to generalize the dataset like this type of road image may come into a real scenario. After this technique, the size of the dataset became more than 12.8K.

### 3.7. Augmentation

Our dataset does not resemble real-world road data, such as gloomy environments, zoomed views, and so on, as we employed a simulator. However, even now, the size of the photograph is insufficient. Augmentation is a procedure that artificially increases a training dataset’s size by modifying the images in the dataset. ImageDataGenerator, a Keras deep learning module, is mostly used in image data augmentation techniques. Among various augmentation techniques, we experimented with four approaches: zooming, panning, brightness, and random flipping that best fit our data.

**Figure 11:**
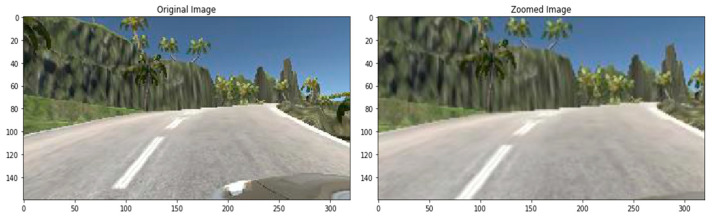
Zoomed image.

In the zooming technique, the image is zoomed randomly by interpolating pixel values or adding new pixel values around the image. If a float is specified, [1−value, 1+value] will be the zoom range. So we do not zoom across the x-axis, but across the y-axis, we zoom at 1.3 scales. Figure 11 is a sample of the zoomed image.

**Figure 12:**
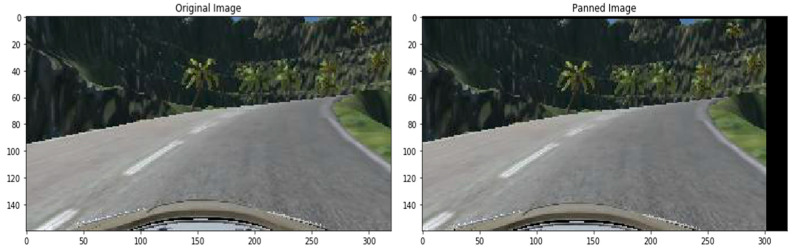
Panned image.

To pan an image, we have selected the following parameters for x and y and a sample image is displayed in Figure 12.

translate_percent = {“x”: (−0.1, 0.1), “y”: (−0.1, 0.1)}

**Figure 13:**
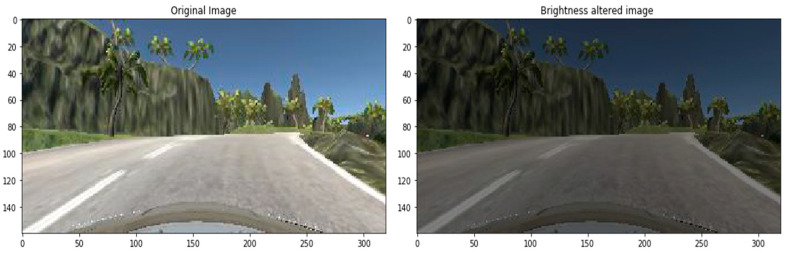
Brightness altered image.

The brightness of the image can be changed either by randomly darkening images, brightening images or both. Values underneath 1.0 obfuscate the image, e.g., [0.5, 1.0], whereas values greater than 1.0 illuminate the object, e.g., [1.0, 1.5] where 1.0 does not affect illumination. We used a scale from 0.2 to 1.2, a sample shown in Figure 13.

**Figure 14:**
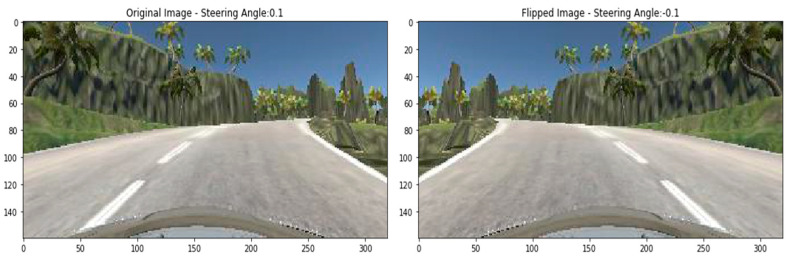
Flipped image.

Flipping into left or right is another technique used in image augmentation. For example, the right-oriented images turned left and left-oriented into right. In previous approaches, we do not need to change the steering angle across the changing of the images. However, in the case of flipping, the road image is the opposite. So, we need to flip the steering angle across the image. A sample flipped image has been displayed in Figure 14 with the flipped steering angle.

### 3.8. Pre-Processing of the Dataset

**Figure 15:**
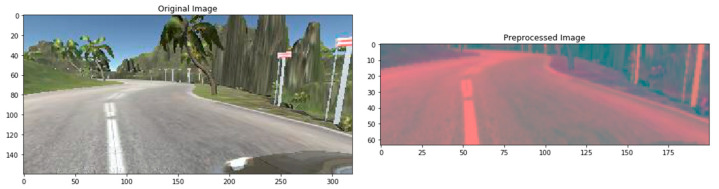
Pre-processed image.

Pre-processing is another crucial technique to smooth the image before feeding it into training steps. We have considered five pre-processing methods. First, an original image and after the pre-processing step, the pre-processed image is shown in Figure 15. From the original image, we have seen that the top part contains natural scenery that do not have any value in steering angle prediction. Besides, removing this part also minimize the size of the image.

The YUV color model is closer to human color perception than the standard RGB model. So, we convert the RGB image into YUV format. Then, blurring the image remove the noise and clean the image. We have used gaussian blur with 3 × 3 kernel size. Then we resize the image into 200 × 64. Finally, to normalize the pixel value, we divide each value via 255 as the value range is 0 to 255 in the original image.

### 3.9. Splitting of the Dataset

**Figure 16:**
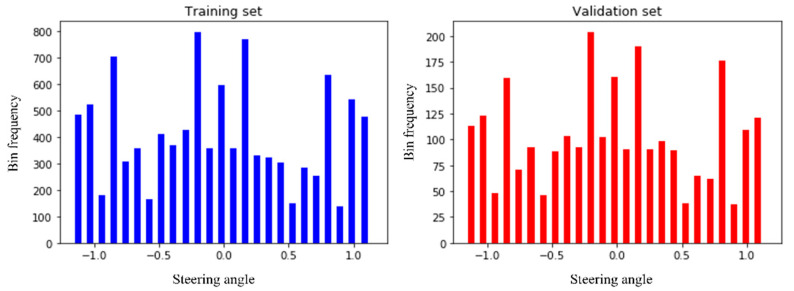
Splitting the images into training and validation set.

After pre-processing, the images need to be split into training and validation sets. The model learns the steering angle through the training set, and via the validation set, it will examine how accurately it learns. We preserve 20% of data for validation purposes so that after training, we can test the performance of the trained model how much it learns. Figure 16 clearly states that the distribution of the training and validation set is quite similar and so fit for the pass into training step.

### 3.10. Convolution Neural Network Architecture

**Figure 17:**
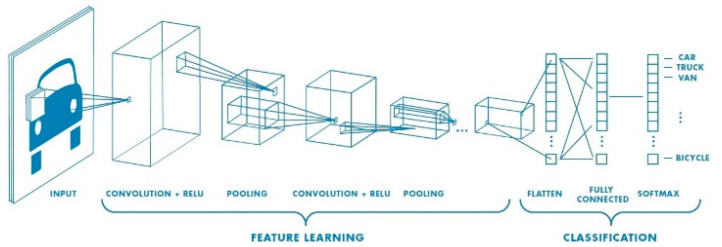
A CNN network with many convolutional layers.

A deep learning Convolution Neural Network (CNN or ConvNet) is a subset of deep neural networks, most commonly used in visual image processing. To train and test deep learning CNN models, each image will go through a sequence of convolution layers with kernels, pooling layer, fully connected layers and apply activation function (softmax, tanh, ReLu etc.) to classify an object with probabilistic values. Figure 17 is a full CNN flow to analyze an image as input and identify the objects according to values.

**Figure 18:**
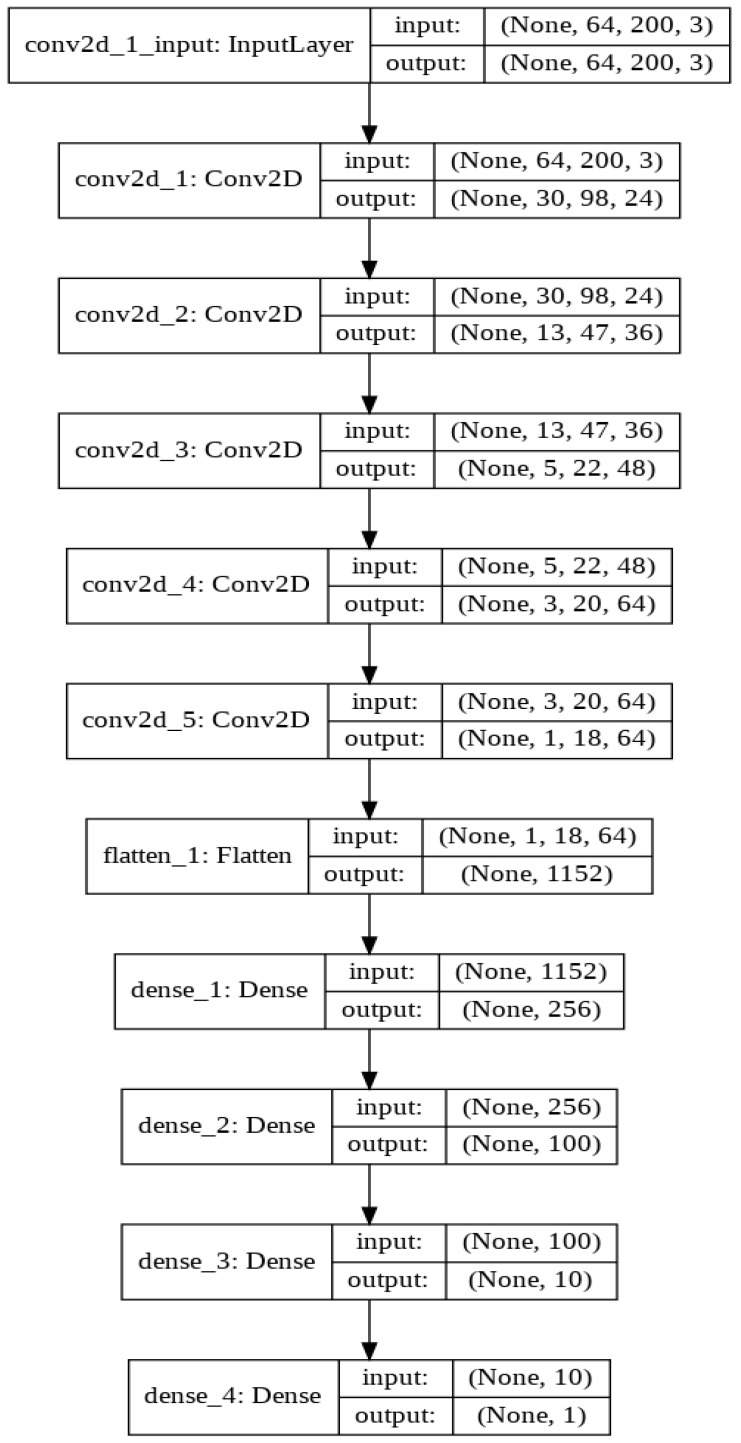
Experimented with Nvidia CNN architecture.

The first layer to extract features from an input image is convolution. Convolution is a dot product of an input image with a kernel to understand the feature. To understand the feature for an image, there have to be various types of convolution kernels. Despite defining the kernel, in CNN, we represent the number of kernels with the dimension. We have followed the Nvidia CNN model to design our deep learning CNN architecture (Figure 18). Nvidia experiments with this CNN architecture in their self-driving project with more than 72-h of video data. They tuned various parameters and found better outcomes for this network. We also found promising results from this architecture. This architecture has nine layers combining five convolution layers, three densely connected layers and one output layer. The first three convolution layers are glued with 2 × 2 subsamples where the first layer, the second layer, third layer have 24, 36, 48 kernels, respectively, with 5 × 5 kernel size. The following two convolution layers include 64 kernels with 3 × 3 size features. Then we flatten the matrix and connect the flatten layer with a dense layer having 256 neurons. The successive two dense layers have 100, 10 neurons accordingly. The final one is the output layer with one neuron. In each layer, we have used ‘elu’ activation function but the output layer. To optimize the model, we have chosen the Adam optimizer. For each deep learning model, the Adam optimization algorithm, an extension to stochastic gradient descent, was used as the optimization algorithm. Recently it has seen broader adoption of computer vision and natural language processing for deep learning applications. 

Because adjusting the parameter learning rates looking at the average initial moments (the mean) as in RMSProp, Adam uses the sum of the gradient’s second moments (the uncentric variance). The algorithm explicitly determines an exponential growth rate of the gradient and the square gradient. The beta1 and beta2 parameters regulate the decay rates of such moving averages. The learning rate used in our model is 0.0001, and the mean squad error is a loss function. Despite fitting all the images into RAM, we have fit the dataset through a batch generator. We run the model with 20 epochs. Then after training, we visualize the loss rate and accuracy rate. If the model loss and accuracy rate are not good, we go back to the pre-processing steps and follow the same flow and again train and visualization. After several times experiment, we have found an optimized model. Then we have saved the model into hdf5 file format, where the network architecture and the weight are stored.

After preparing the trained model, we have tested the model into a Udacity simulator in an autonomous model where the model is fitted with the simulator. The input image fits the model after pre-processing steps and predicts the steering angle following which the car moves forward. We have developed a prototype to experiment with how the self-driving car model works in real life from the perspective of Bangladesh. We assemble the hardware parts, including 4-wheel chassis board, 4 motors, L298 motor driver, Lipo battery, buck converter, raspberry pi, camera, power switch, etc.

### 3.11. Driving Instruction through L298 Motor Driver

Four motors are connected with four wheels, and the L298 motor driver controls the direction and rotation of the motor. Battery power is distributed to the L298 motor driver and raspberry pi with a buck converter and a USB cable. A Noir Camera is placed in front of the camera and directly attached to the raspberry pi. The camera module captures the road video and passes the image to the raspberry pi at a 10 fps rate. The raspberry pi passes the image to the pc through server communication on TCP protocol. The pretrained model takes the image as input, predicts the steering angle, and transfers it back to the raspberry pi. Based on the steering angle, the raspberry pi commands the L298 motor driver to move accordingly.

**Figure 19:**
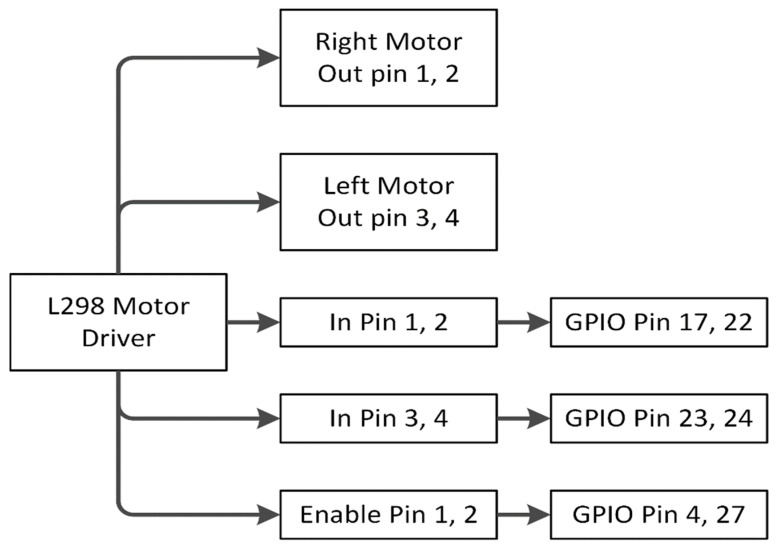
L298 Motor driver working process.

The working procedure of the L298 motor driver is shown in Figure 19. The out pin 1, 2 is connected with the right motor and pin 3, 4 with the left motor. Enable pin 1 for the right motor that is connected with GPIO pin 4 of the raspberry pi. Similarly, enable pin 2 for the left motor with GPIO pin 27. Input pin 1, 2 of the L298 driver is connected with GPIO 17, 22 for the right motor and GPIO 23, 24 with input pin 3, 4 for the left motor.

## 4. Experimental Result

**Figure 20:**
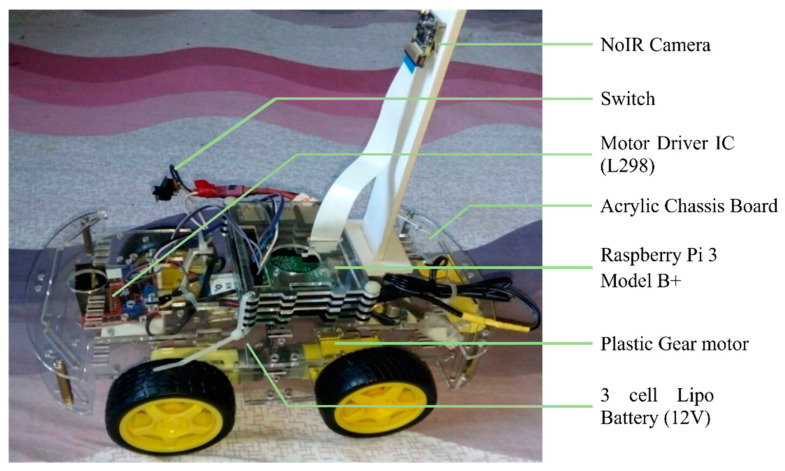
Hardware assembly of self-driving car.

We have developed a prototype to experiment with how the self-driving car model works in real life from the perspective of Bangladesh (Figure 20). First, we assemble the hardware parts, including 4-wheel chassis board, 4 motors, L298 motor driver, Lipo battery, buck converter, raspberry pi, camera, power switch etc. Then, four motors are connected with four wheels, and the L298 motor driver controls the direction and rotation of the motor. Finally, battery power is distributed to the L298 motor driver and raspberry pi with a buck converter and a USB cable.

**Figure 21:**
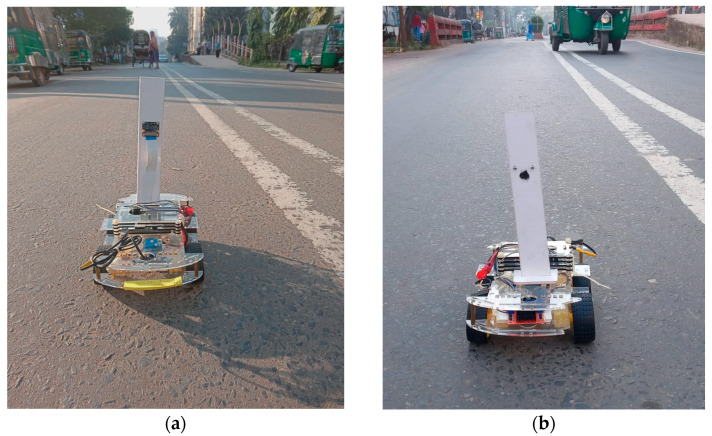
The performance of the car in the various environment (**a**) in lightening atmosphere (**b**) in a cloudy atmosphere.

The performance of the car is tested on the actual road track. The vehicle is tested in both lightening and cloudy atmospheres to understand its behavior on the change of the environment. Figure 21a represents the lightening environment, and Figure 21b a little bit of a cloudy environment. In both environments, the car performs very well to maintain its track on the actual road.

**Figure 22:**
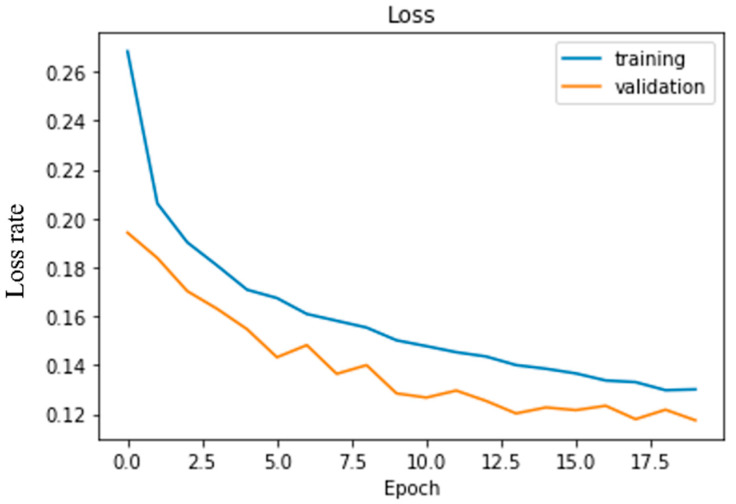
The loss rate of experimented CNN architecture.

The Noir camera module is placed in front of the camera and directly attached to the raspberry pi. The camera module captures the road video and passes the image to the raspberry pi at a 10 fps rate. The raspberry pi passes the image to the pc through server communication on TCP protocol. The pretrained model takes the image as input, predicts the steering angle, and transfers it to the raspberry pi. The car moves towards its direction based upon the steering angle. The loss rate of the deep learning CNN model is shown in Figure 22. From this scenery, we have seen that the loss rate is decreasing for both training and validation datasets regarding increasing the number of epochs. Validation loss and training loss difference is very well. Therefore, the model is neither overfitted nor underfit.

The accuracy of the model is measured in various environments or turning. Table 2 lists all the accuracy where on lightening conditions the model outperforms then cloudy climate. Similarly, right turning accuracy is 89.3% higher compared to straight and left turning.

In terms of accuracy, we compared our model to the previous literature in Table 3 as well. The temporal fusion process employed in the TCNN setup is temporal convolution. A fixed-length window of three (TCNN3) and nine (TCNN9) seconds was used. The performance of TCNN models continues to increase, and the larger the time horizon, the better. That’s why TCNN9 accuracy is 84.6% better than TCNN3 83.3%. However, it needs a fixed size history window and is more memory intensive than the LSTM-based method. It performs similarly 84.5% to TCNN9 when using the CNN-LSTM method. While the Nvidia CNN architecture studied in our research shows an overall 89.2% that is notable than other configurations.

**Figure 23:**
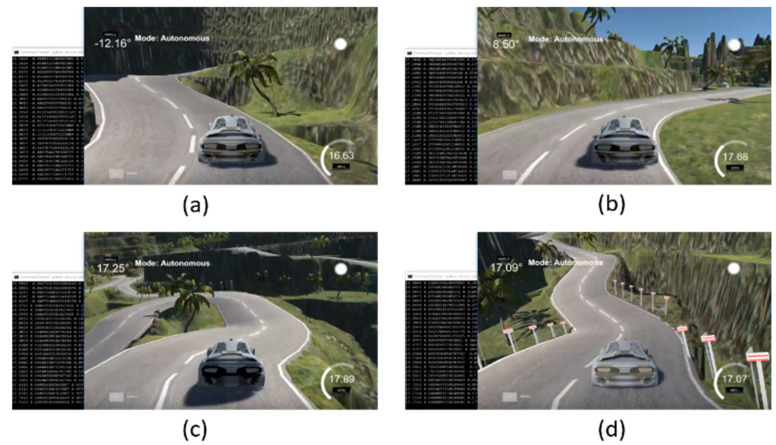
Performance of the model into autonomous mode (**a**) left turn prediction angle (**b**) right turn prediction angle (**c**) complex right turn prediction angle (**d**) hill tracked right turn prediction angle.

The performance of the whole autonomous product delivery car network is recorded on a per-frame basis. The camera sensor on the car passes 10 frames per second to the remotely connected high configuration pc via raspberry pi that requires 0.07 sec per frame. The image processing and to be predicted the steering angle requires 0.02 sec per frame. The steering angle info then sent back to the raspberry pi to drive the car accordingly requires another 0.03 sec. The network requires 0.12 sec per frame from image capture to prediction. The performance of the trained model is experimented with using the Udacity simulator in autonomous mode. A few snapshots from the various angle in autonomous mode have been demonstrated in Figure 23. Through socket programming, the Udacity simulator passes the road image to the model and predicts the steering angle. This steering angle back to the car, and the vehicle moves according to that angle. The predicted steering angle is shown in the top-left position of the Udacity simulator. Figure 23a is a sample for a left turn where we have found that the model predicts angle as −12.16° and Figure 23b another rightly turned position and model predict 8.50° steering angle. More curved situations are also displayed in Figure 23c, complex right turn, and 23d, hill tracked right turn, where the model predicts 17.25° and 17.09°, respectively. Furthermore, the predicted steering angle is shown in the command prompt at the left position of the images.

The visualization of the geographical position of the vehicle is one of the great features to track the car immediately. At the time of product delivery, the vehicle owner can track his vehicle at any time. Geoip2 library is used to track the car from its IP address.

**Figure 24:**
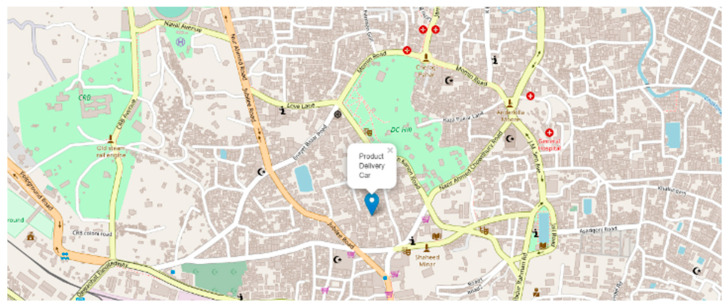
Map visualization of the geographical position of the car.

After being given the IP address, geoip2 returns the geographical data of that vehicle. Those geographical data, i.e., longitude and latitude, are visualized through the Folium library of Python programming language. A demonstration of the current position of the self-driving product delivery vehicle is shown in Figure 24.

**Figure 25:**
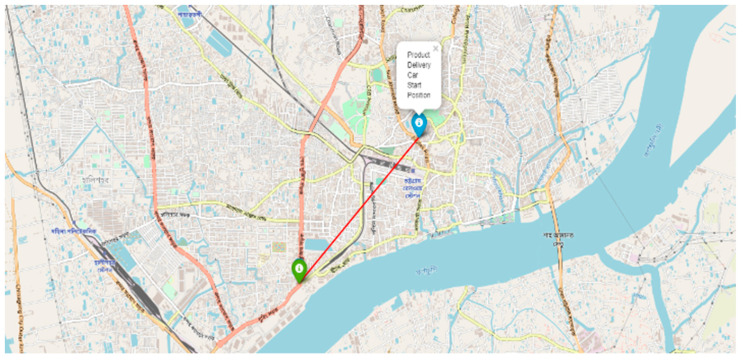
Map visualization of the source and destination place of the car.

**Figure 26:**
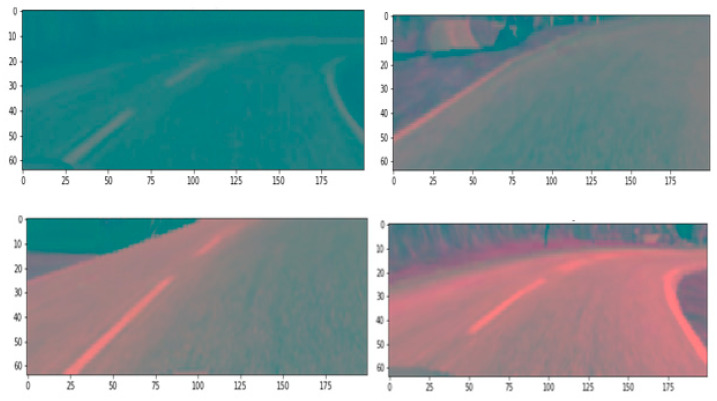
Sent Pre-processed image at testing time of the vehicle.

From the perspective of Bangladesh Road, the model is tested in several environments such as darkness, brightness distortion, gloomy atmosphere, etc., and performs at a satisfactory level. A source to destination position is shown in Figure 25. Because of the diverse environment augmentation to the original dataset, the vehicle is fit for the actual road of Bangladesh. We have experimented with the car at the Chittagong—Cox’s Bazar highway at the Rahattarpul area. During the self-driving vehicle movement, we have stored several pre-processed images that are sent from the Raspberry pi attached camera module shown in Figure 26.

## 5. Discussion and Conclusions

There is a lot of trouble with on-time product delivery from Bangladesh’s perspective, and human decision-making errors cause severe road accidents. Many drivers obey their feelings even though they are not correct in the moment. Thus, driving system automation will solve those problems. Therefore, autonomous vehicles can ensure on-time product delivery and reduce accident rates because of human error. We have developed Delicar, such a low-cost self-driving product delivery vehicle where the camera placed on the roof of the vehicle capture the image and raspberry pi sends the image to the pre-trained model for steering angle with respect to that image.

Moreover, it is low in cost and easy to implement. However, there does not exist any authentication system to receive the product. Anyone from the destination can receive the product that is a shortcoming of the study. Extensive chances of development in this work are kept open. Lots of essential features can be added to it in the future. To detect damages and holes in preceding the vehicles in the road using cameras and sensors and produce warning system is the future scope of our research along with double step authentication to receive the product such as password, fingerprint, etc. The future direction of the study also includes the most effective path programming and obstacle avoidance to reach the destination safely and quickly. The interplay of smart people, smart technology, and smart processes, which may be shown as the Smart Golden Triangle, eventually determines the success of smart cities. Such an intelligent product delivery car will drive the smart city to the next level. However, in Bangladesh, the traffic congestion costs 3.2 million working hours daily, BDT 200 billion annually. To ensure the traffic rules are followed and strictly avoid overtaking, the self-driving car is a great alternative. As an impact of such a solution, self-driving product delivery cars will contribute to the economy via utilizing very few human resources. This autonomous product delivery car will advance the e-commerce industry to the next level by ensuring on-time delivery. In supply chain management, self-driving product delivery cars may not only have a significant influence on logistics by lowering costs and delays, but they could also have a significant impact on distribution and manufacturing centers. Therefore, the Government should instantly install this proposed system to deliver the product in time to save Bangladesh’s economic deterioration.

## Figures and Tables

**Table 1:** Recorded data at training mode.

Center	Left	Right	Steering	Throttle	Reverse	Speed
E:\android\curly_final\IMG\center_2019_04_12_01_52_50_770.jpg	E:\android\curly_final\IMG\left_2019_04_12_01_52_50_770.jpg	E:\android\curly_final\IMG\right_2019_04_12_01_52_50_770.jpg	0	0	0	0.00014
E:\android\curly_final\IMG\center_2019_04_12_01_52_50_846.jpg	E:\android\curly_final\IMG\left_2019_04_12_01_52_50_846.jpg	E:\android\curly_final\IMG\right_2019_04_12_01_52_50_846.jpg	0	0	0	0.000199
E:\android\curly_final\IMG\center_2019_04_12_01_52_50_917.jpg	E:\android\curly_final\IMG\left_2019_04_12_01_52_50_917.jpg	E:\android\curly_final\IMG\right_2019_04_12_01_52_50_917.jpg	0	0	0	0.00026

**Table 2:** Performance of the model on various environment/turn.

Environment/Turning	Accuracy
Cloudy	88.9%
Lightening	89.6%
Left	87.1%
Right	89.3%
Straight	89.0%

**Table 3:** Accuracy comparison with previous literature proposed architecture.

Configuration	Accuracy
TCNN3	83.3%
TCNN9	84.6%
CNN-LSTM	84.5%
Nvidia CNN	89.2%

## Data Availability

Data is describing within the article. The data that support the findings of this study are available from the corresponding author upon reasonable request.
